# Laparoscopic Resection of an Appendix Mucocele in a Breast Cancer Patient

**DOI:** 10.1155/2018/1780342

**Published:** 2018-10-09

**Authors:** Scarlett Hao, Roberta Lilly, Hugo J. R. Bonatti

**Affiliations:** ^1^Vidant Medical Center, Greenville, NC, USA; ^2^University of Maryland Community Medical Group Surgical Care, Easton, MD, USA; ^3^Meritus Surgical Specialists, Hagerstown, MD, USA

## Abstract

**Background:**

Acute appendicitis may be treated with antibiotics, but most surgeons offer laparoscopic appendectomy (LA). Appendiceal mucocele (AMC) is a rare disorder. Surgical removal is recommended due to the risk of pseudomyxoma peritonei. LA has been suggested for this condition. Although rare, breast cancer (BC) may metastasize to the appendix. An appendiceal mass in a breast cancer patient should be approached as a possible metastatic focus until proven otherwise.

**Case Presentation:**

A 45-year-old Caucasian woman with invasive lobular BC underwent bilateral mastectomy. An AMC was found on CT scan. LA was done with a strict minimal touch technique. The appendix was resected with a 1 cm margin of the cecal pole, and the specimen was removed from the abdomen in a retrieval bag. Pathology showed benign cystadenoma. The patient had an uneventful postoperative course.

**Conclusion:**

This case highlights the diagnostic challenge of an appendiceal mass in a BC patient. BC patients with AMC should undergo appendectomy to rule out metastatic disease and to prevent pseudomyxoma peritonei. LA can be performed safely in patients with AMC.

## 1. Introduction

Acute appendicitis (AA) may be treated with antibiotics; however, laparoscopic appendectomy (LA) is still preferred by most surgeons [1, 2]. In appendix mucocele (AMC), the appendix lumen is filled with mucus. This rare condition may clinically mimic AA; however, many patients are asymptomatic, and diagnosis is made incidentally on ultrasound or CT scan [3]. Cystic luminal distention secondary to obstruction by a mucinous cystadenoma or cystadenocarcinoma causes the majority of AMC [3]. If neoplastic AMC ruptures, pseudomyxoma peritonei (PMP) may develop [4]. BC may metastasize to the appendix mimicking AA and may even perforate [5, 6] but commonly is found incidentally [7–9].

Due to the potential of harboring malignancies, AMCs should be removed. LA is feasible and safe if certain operative principles are followed.

## 2. Case Report

**Figure 1:**
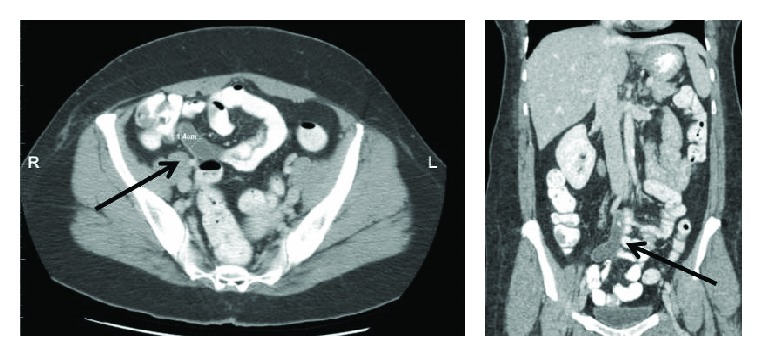
CT scan: appendix mucocele (arrow).

A 45-year-old BRCA-negative Caucasian female presented with a self-detected breast mass and signs of breast dimpling. Imaging demonstrated a 9.5 cm breast mass with biopsy returning a finding of ER/PR positive invasive lobular carcinoma of intermediate grade. She underwent uneventful bilateral mastectomy revealing invasive lobular BC involving one out of 12 axillary lymph nodes; stage IIIA T3 N1. Bone scan prior to adjuvant chemotherapy was negative. She had no acute abdominal pain but reported some recurrent abdominal discomfort during the past year. Therefore, a CT scan was done, which demonstrated a significantly enlarged appendix without stranding or inflammation indicating AMC (Figure 1). Appendiceal carcinoma or BC metastasis could not be ruled out. The patient had a normal WBC. Possible appendix malignancy and risk to develop AA during chemotherapy were discussed, and consent for LA was obtained.

**Figure 2:**
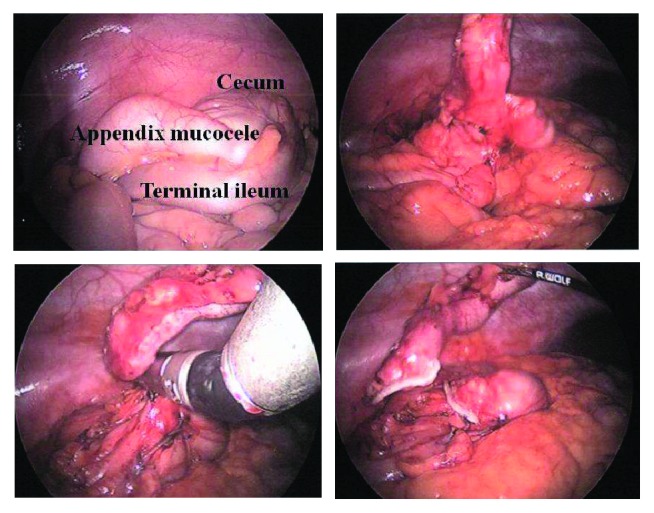
Operative findings: left upper: overview of the right lower quadrant showing appendix mucocele; right upper: the appendix is gently lifted up; left lower: the appendix is stapled just below the base; right lower: the appendix has been stapled, only the mesoappendix is grabbed.

5 mm trocars were placed in the left upper and lower quadrant and a 10–12 mm trocar into the umbilicus. The AMC was gently lifted up (Figure 2); at no point, the appendix was grasped. A window was created behind the AMC, and the mesoappendix was stapled. The cecal pole was mobilized, and the AMC was stapled off with a 1 cm rim of cecal wall (Figure 2) avoiding stenosis of the terminal ileum. The specimen was immediately placed into an endobag and removed from the abdomen via the dilated umbilical port. The postoperative course was uneventful. Histopathology revealed a benign cystadenoma with clean margins and no rupture. Chemotherapy for her BC was started four weeks later. Bilateral salpingo-oophorectomy was done a year later. The patient is alive and well without tumor recurrence and without intraperitoneal symptoms after 3 years.

## 3. Discussion

Prophylactic appendectomy in subsets of BC patients has been suggested [7, 10]. Risk-reduction appendectomy with bilateral salpingo-oophorectomy in *BRCA1* carriers could lead to a 99% reduction in lifetime risk of intraperitoneal cancer [10]. Although BC metastases to the appendix are rare, prophylactic appendectomy at the same time as prophylactic salpingo-oophorectomy may be warranted [7]. In our patient, a CT scan was done showing the AMC, and we opted for LC and salpingo-oophorectomy at a later stage.

Benign and malignant AMC may mimic AA but also adnexal masses [11, 12]. The risk of spillage leading to PMP prohibits the use of needle biopsy [12]. Receptor status and histologic type of BC may predict gastrointestinal metastases; ER positive and negative and ductal and lobular BC may cause appendix metastases [9, 13]. Concurrent medication (corticosteroids, analgesia, and chemotherapy) may mask symptoms and delay diagnosis [7].

In 1998, Gonzalez Moreno et al. still advocated that AMC is a contraindication for laparoscopic appendectomy based on a case in which spillage caused secondary PMP [14]. However, since 2000, multiple cases and case series demonstrated that LA for mucinous cystadenoma shows excellent outcomes including negative margins and minimal hospital stay without subsequent PMP or need for conversion to open surgery [11]. Orcutt et al. reported hand-assisted and robotic-assisted appendectomy; however, conventional laparoscopic appendectomy as in our case and shown in the large series by Park et al. seems equally safe with much lower costs [11, 15]. The AMC must be handled carefully without grasping; a safety margin including a rim of cecal pole is required, and the specimen must be retrieved in a bag; any spillage must be avoided [11]. Wrapping the AMC with gauze has recently been suggested and has become an accepted technique [16].

Patients with AMC including those with BC should have their appendix removed. LA is safe and feasible.

## References

[1] Li X., Zhang J., Sang L. (2010). Laparoscopic versus conventional appendectomy--a meta-analysis of randomized controlled trials. *BMC Gastroenterology*.

[2] Rollins K. E., Varadhan K. K., Neal K. R., Lobo D. N. (2016). Antibiotics versus appendicectomy for the treatment of uncomplicated acute appendicitis: an updated meta-analysis of randomised controlled trials. *World Journal of Surgery*.

[3] Dixit A., Robertson J. H., Mudan S. S., Akle C. (2007). Appendiceal mucocoeles and pseudomyxoma peritonei. *World Journal of Gastroenterology*.

[4] Buell-Gutbrod R., Gwin K. (2013). Pathologic diagnosis, origin, and natural history of pseudomyxoma peritonei. *American Society of Clinical Oncology Educational Book*.

[5] Burney R. E., Koss N., Goldenberg I. S. (1974). Acute appendicitis secondary to metastatic carcinoma of the breast. A report and review of two cases. *Archives of Surgery*.

[6] Capper R. S., Cheek J. H. (1956). Acute appendicitis secondary to metastatic carcinoma of the breast. *AMA Archives of Surgery*.

[7] Maddox P. R. (1990). Acute appendicitis secondary to metastatic carcinoma of the breast. *The British Journal of Clinical Practice*.

[8] Molina-Barea R., Rios-Peregrina R. M., Slim M., Calandre E. P., Hernandez-Garcia M. D., Jimenez-Rios J. A. (2014). Lobular breast cancer metastasis to the colon, the appendix and the gallbladder. *Breast Care*.

[9] Mori R., Futamura M., Morimitsu K., Yoshida K. (2016). Appendicitis caused by the metastasis of HER2-positive breast cancer. *Surgical Case Reports*.

[10] Sitzmann J. V., Wiebke E. A. (2013). Risk-reducing appendectomy and the elimination of BRCA1-associated intraperitoneal cancer. *JAMA Surgery*.

[11] Park K. J., Choi H. J., Kim S. H. (2015). Laparoscopic approach to mucocele of appendiceal mucinous cystadenoma: feasibility and short-term outcomes in 24 consecutive cases. *Surgical Endoscopy*.

[12] Rabie M. E., al Shraim M., al Skaini M. S. (2015). Mucus containing cystic lesions “mucocele” of the appendix: the unresolved issues. *International Journal of Surgical Oncology*.

[13] Kwan E., Houli N., Pitcher M., Wong S. (2016). Appendiceal metastasis in a patient with advanced breast cancer on hormonal therapy. *International Journal of Cancer and Clinical Research*.

[14] Gonzalez Moreno S., Shmookler B. M., Sugarbaker P. H. (1998). Appendiceal mucocele. Contraindication to laparoscopic appendectomy. *Surgical Endoscopy*.

[15] Orcutt S. T., Anaya D. A., Malafa M. (2017). Minimally invasive appendectomy for resection of appendiceal mucocele: case series and review of the literature. *International Journal of Surgery Case Reports*.

[16] Yoshida Y., Sato K., Tada T. (2013). Two cases of mucinous cystadenoma of the appendix successfully treated by laparoscopy. *Case Reports in Gastroenterology*.

